# Efficacy of the self-mutual-group model targeting quality of life improvement among empty-nest older adults in Taiyuan, China: an intervention study

**DOI:** 10.1186/s12877-021-02155-4

**Published:** 2021-03-25

**Authors:** Chichen Zhang, Yaqing Xue, Yuan Cai, Jiao Lu, Xiao Zheng, Wenpei Yuan, Yi Qian

**Affiliations:** 1 School of Health Services Management, Southern Medical University, No. 1023, Shatai South Road, Guangzhou, 510515 Guangdong China; 2 Department of Health Management, Nanfang Hospital, Southern Medical University, Guangzhou, 510515 Guangdong China; 3 Institute of Health Management, Southern Medical University, Guangzhou, 510515 Guangdong China; 4 School of Public Health, Southern Medical University, Guangzhou, 510515 Guangdong China; 5 School of Management, Shanxi Medical University, Taiyuan, 030001 Shanxi China

**Keywords:** Intervention study, Empty-nest older adults, Quality of life, Health management model

## Abstract

**Background:**

In China, more and more older people have encountered a situation called “empty nest.” Meanwhile, the health status of empty-nest older adults is an increasing public health concern. This research aims to examine the effectiveness of Self-Mutual-Group (SMG) model in improving quality of life of the empty-nest older adults to provide a scientific evidence for improving their health.

**Methods:**

A prospective intervention study was conducted among empty-nest older adults in Taiyuan, Shanxi. Multi-stage stratified random cluster sampling was employed to selected participants. A total of 396 empty nesters were enrolled as participants, of which 204 and 192 were in the intervention and control group, respectively. The intervention group received a seven-month SMG-based intervention. A participant’s quality of life was measured at the baseline and seven months after using the Short Form 36-Item Health Survey (SF-36).

**Results:**

No significant difference was found between the intervention and control groups in terms of participant characteristics at baseline (*P* > 0.05). After the intervention, participants’ scores on Mental Component Summary (MCS), Physical Component Summary (PCS), role emotional (RE), vitality (VT), social function (SF), mental health (MH) and general health (GH) increased significantly in the intervention group. Additionally, these scores differed significantly from those in the control group (*P* < 0.05).

**Conclusion:**

This study showed that the SMG-based health management is effective in improving quality of life among empty-nest older adults after seven months.

**Trial registration:**

Study on the ‘SMG’ Health Management Model Based on Community Organization Theory among empty-nest older adults (ChiCTR1800015884). Registration date: 26-04-2018. Retrospectively registered.

**Supplementary Information:**

The online version contains supplementary material available at 10.1186/s12877-021-02155-4.

## Background

With rapid economic development, the extension of average life expectancy, and the decrease in fertility rate, China has entered a period of accelerated population aging. Currently, on the mainland China, approximately one fifth of the total population is aged 60 years and older, accounting for more than 200 million people [1]. At the same time, more older people have encountered a situation called “empty nest.” The empty nest families refer to an older adult have no children or whose children have already left home [2]. In the traditional family pattern, which is influenced by the Confucian filial piety culture, children usually take care of their parents when the latter get older. In recent years, however, because of practical problems, such as work and family, an increasing number of children choose not to live with their parents. This phenomenon means that often no adult children is around when older adults need help. In 2014, a report published by China National Committee on Aging showed that the proportion of older people living alone was rising. By 2020, the number of empty nesters will grow to 118 million [3], the increasing number of empty nesters will bring great challenges to the health care system and become a public health concern.

Healthy aging is manifested in not only the extension of the life of the older people but also, more importantly, the improvement of their quality of life [4]. However, the overall health condition of older adults is not optimistic. Studies have showed that with the growth of age, the physical health, self-care ability and cognitive function of the older adults tend to decline [5, 6]. A systematic review demonstrated that among older Chinese adults (aged ≥60 years), the overall prevalence of multimorbidity was up to 87.0% in urban residents [7]. As a vulnerable group, shrinking social resources and a lack of emotional support for empty nesters could lead to a greater risk of health problems [8]. Many studies that have been conducted in China demonstrated that the social supports, quality of life, and mental health were poor in empty-nest older adults. A research [9] found that in Xiamen City, empty nesters had lower values on a range of indicators relating to physical and mental health and social adaptation; moreover, older adults living alone had a significantly worse quality of life than others. A significant association was also found among empty nesters, noting that they are more inclined to report long-standing illness when compared to the non-empty-nest group [10]. Some studies also found that empty-nest older adults may experience more discomfort, anxiety, and depression [11]. Gao et al. found that empty nest had a significant adverse influence on older people’ physical health, cognitive ability, and psychological health [12]. In fact, empty nesters have experienced not only the restructuring of their lifecycle but also the transformation of the family cycle. When children move out of their homes, the older people usually feel abandoned by their children and become more frustrated, depressed, and anxious. Therefore, it is necessary to find effective ways to improve the quality of life of empty-nest older adults.

Previous studies have proposed various interventions to improve the quality of life of the older adults but showed limited effectiveness. Van Uffelen et al. [13] conducted a randomized, placebo controlled intervention trial to examine the effect of walking and vitamin B supplements on quality of life. The results showed that the walking program and vitamin B supplements were not effective in improving quality of life in community-dwelling older adults. Taguchi et al. [14] found that although several studies have shown beneficial effects of exercise on health-related quality of life (HRQOL) in the older adults, their intervention trial failed to demonstrate such an effect for any measure of HRQOL, except for the Falls Efficacy Scale. The limited effectiveness of interventions in these studies may be related to participants’ compliance and motivation. A few researchers also found that participants’ motivation was not high during the intervention process, and the interventions were only maintained until the end of the study [15]. Thus, some researchers have tried to conduct group interventions. Wu et al. [16] conducted a group psychological intervention to improve quality of life among cancer patients, showing significant changes in quality of life. Wang et al. [17] found that community health management can improve blood pressure control and the quality of life for senior hypertensive patients. The group intervention can take measures against common goals, which helps to enhance respondents’ compliance. However, this intervention cannot meet all specific individual requirements. For example, in the survey, we found that for aged patients with diabetes, different individuals have special situations. Part of the older adults have dawn phenomenon, that is, blood sugar increased before breakfast. Therefore, effective interventions should not only consider individual needs but also help participants maintain long-term behavioral changes to achieve long-term benefits.

Based on the above, we focused on improving individuals’ awareness of health management and ability in self-management, mutual-management, and group-management by increasing their self-efficacy, and then constructed a Self-Mutual-Group (SMG) model [18]. Considering the particularity of the empty-nest older adults, we divided SMG-based health management into three stages and designed a set of interventions for individuals and groups. The first stage involves the provision of individual health management guidance according to the health needs of each participant. In the next two stages, peers and team members play a major role. Encouragement from peers and members can inspire participants’ confidence and determination for continuous participation. We hypothesized that the SMG-based intervention is effective in improving quality of life of the empty-nest older adults. The purpose of this study was to conduct an empirical study of the empty nesters in Shanxi Province to evaluate the effects of the SMG-based health management model on quality of life.

## Methods

### Study design and participants

From October 2016 to May 2017, a randomized controlled trial was conducted in Shanxi province, located in northern China. A multi-stage stratified random cluster sampling method was used to select participants. The study was approved by the institutional review boards of Shanxi Medical University, which was registered in a clinical trial registry (ChiCTR1800015884). A written informed consent was obtained from all participants.

**Fig. 1:**
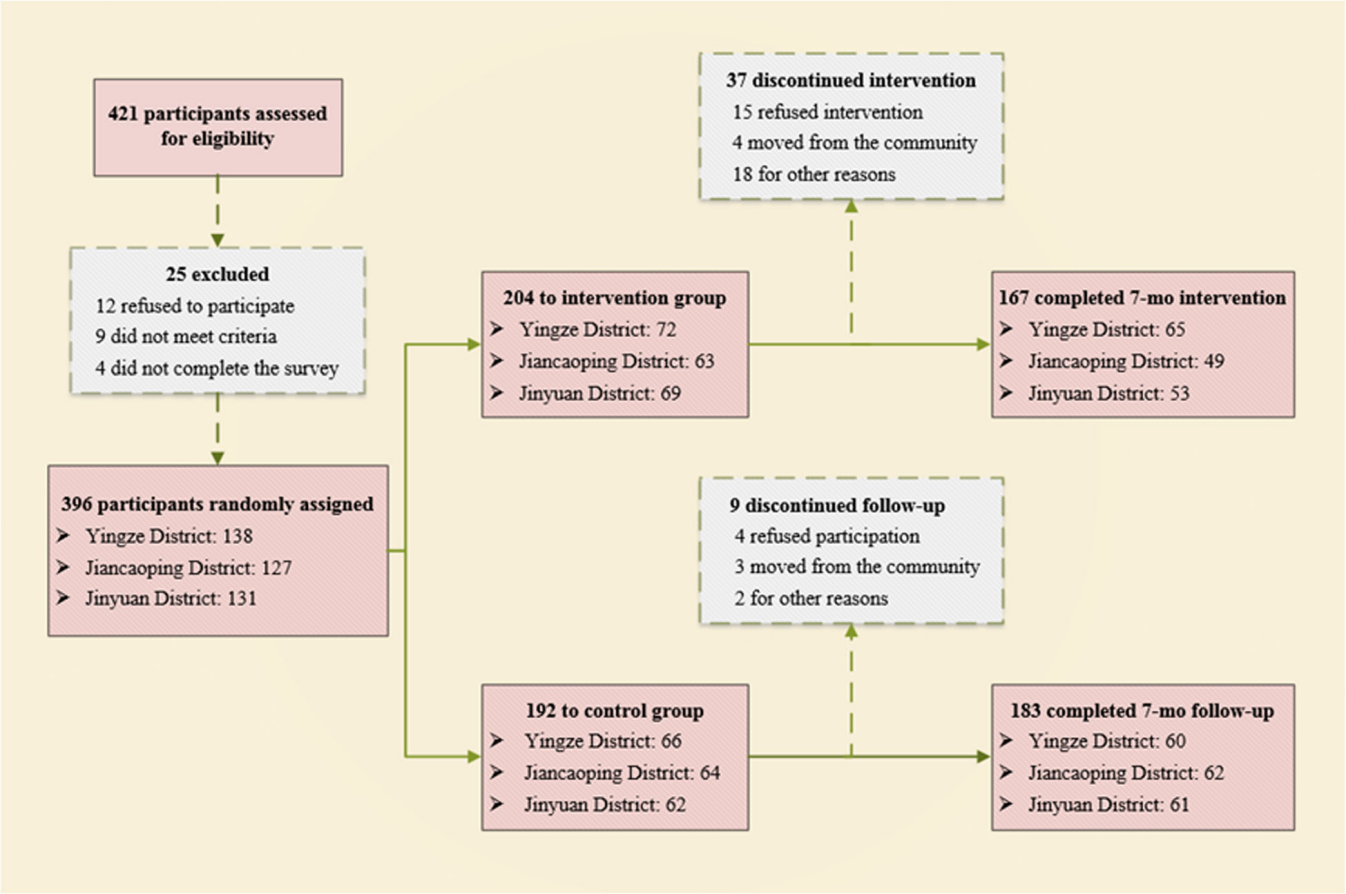
Trial profile

Taiyuan is the capital of the Shanxi Province and has six districts. In the first stage, according to the gross domestic product (GDP) based on the government’s website, the three districts Yingze (high economic level), Jiancaoping (medium economic level), and Jinyuan (low economic level) were randomly selected as the study sites. In the Second stage, each community in the three selected districts was numbered according to the order of communities in the government website, then two communities were selected randomly in each district using a random-number table. Finally, all the empty-nest older adults living in the selected communities were considered candidates for participation in the study. Inclusion criteria were aged greater than or equal to 60 years, who had no cognitive disorder or other mental illnesses, provided informed consent, were willing and able to complete the investigation, and were residing in the community for at least a year before the study. The exclusion criterion was having cognitive disorders or serious diseases, such as deafness, psychiatric disorders, or Alzheimer’s disease. The sample size was estimated according to the following formula *N*_*1*_ *= N*_*2*_*=* $\frac{2{\left({Z}_{\alpha }+{Z}_{\beta}\right)}^2\times {\sigma}^2}{d^2}$. *d*: Mean_1_-Mean_2._ (Mean_1_, the mean of SF-36 score among intervention group, Mean_2_, the mean of SF-36 score among control group), *σ*: the standard deviation of control group. According to the pretest survey, *d* = 6.77, *σ* = 15.69, *α* = 0.05, Z_*α*_ = 1.96, *β* = 0.1, Z_*β*_ = 1.282. The sample size was determined to be 112. An additional 10% was added to this sample estimate in anticipation that the final sample would include individuals who would not consent to participate in the survey. Thus, the final sample size was estimated to be 150 at least. Factually, a total of 396 empty nesters were enrolled as participants, of which 204 and 192 were in the intervention and control group, respectively. Figure 1 presents the details of participant involvement.

### Interventions

**Fig. 2:**
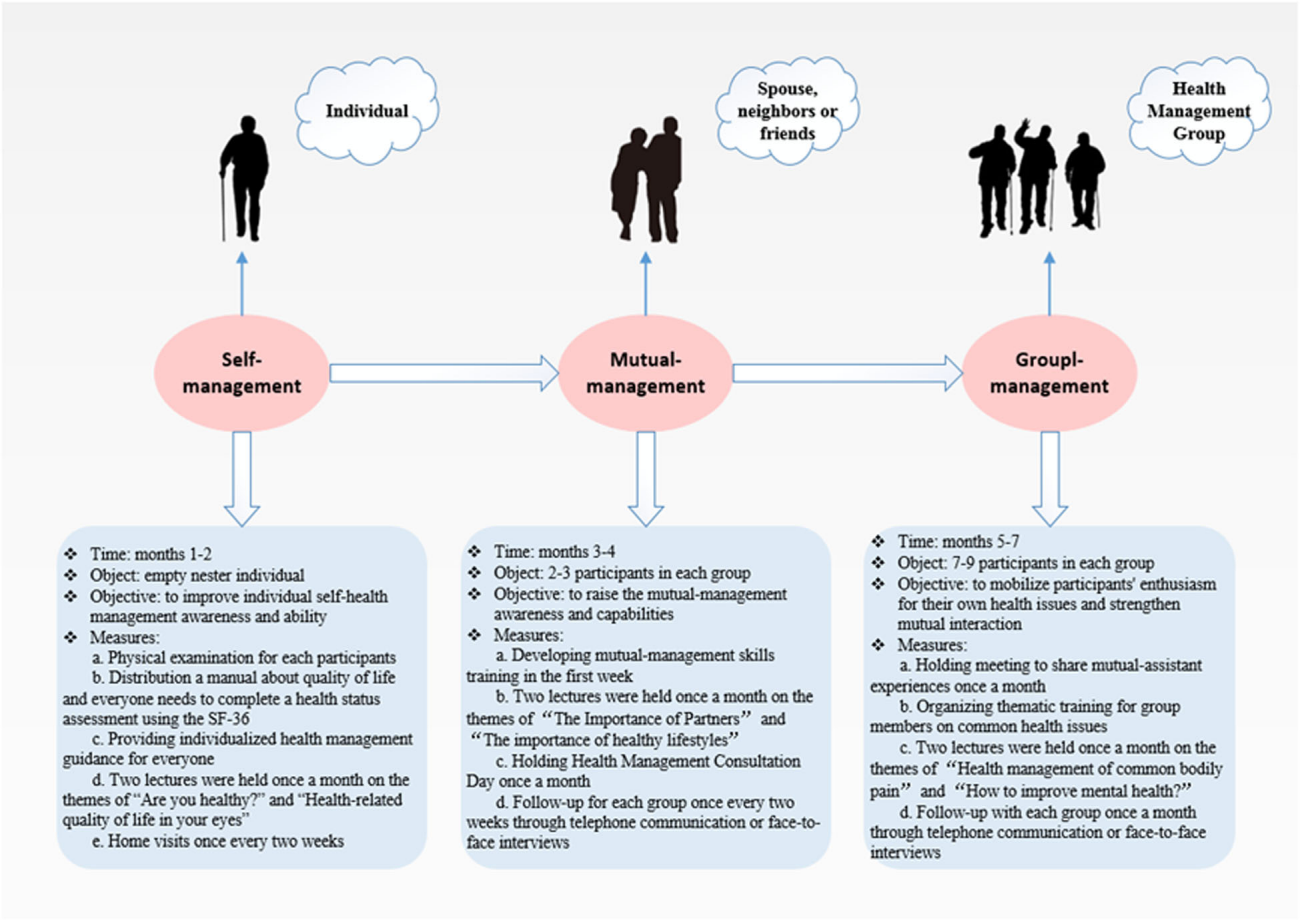
Logic model for the intervention on quality of life improvement based on the SMG model

The intervention group participated in a seven-month SMG-based intervention, which consisted of three stages: self-management (2 months), mutual-management (2 months), and group-management (3 months). The first stage (self-management) was aimed at the empty-nest older adults individuals to develop their self-health management awareness and ability, such as self-care awareness, active medical awareness, self-health assessment ability, and self-service medical equipment use ability. At mutual-management stage, the empty nesters in the same community were paired according to their age, sex, relationship status, home distance, and other factors to form mutual-management; if necessary, volunteers or study staff members were introduced to participate in the pairing. At this stage, empty nesters can share their experiences and knowledge with each other and provide necessary help through life or emotional support. The third stage involved the implementation of group health management based on the first two levels. Groups were categorized by disease type and residential area. For the former, because the empty-nest older adults often face common health problems, there is a common interest in implementing certain goals. For the latter, the principle of proximity was considered; participants were expected not to drop out of the intervention because of distance problems. Group interventions provided an arena within which participants can both provide and receive support. Details of the intervention are shown in Fig. 2. In the entire intervention process, researchers and community workers played the role of health management instructors to assist in the implementation and development of the three types of management. The control group received a routine follow-up without any intervention. Finally, 350 empty-nest older adults completed the study continuously, of which 167 and 183 completed the seven-month SMG-based intervention and seven-month routine follow-up, respectively. Attrition rate was 11.6%.

### Instruments

All participants were invited to complete a self-made standardized questionnaire (Additional file 1). The questionnaire consists of two sections. The general information questionnaire was used to assess the participants’ demographic information, including sex (male, female), age (60–69, 70–69, 80 and above), education (no education, primary school, secondary school, high school, junior school, and university and above), marital status (married, never married, divorced and widowed), employment status (working, and not working), monthly income (no income, less than 1000 RMB, 1000–3000 RMB, more than 3000RMB), spousal relationship, relationship with children, social activity participation, self-care ability, and chronic disease. Relationship with spousal and children was measured through the following question, “How is your relationship with your spouse/children?” Possible answers were “perfect”, “good”, “bad”, “worst”. Social activities such as square dance, playing chess, and traveling were measured through the question, “How do you rate your participation in social activities?” Possible answers were, “most”, “more”, “less” and “no.” Self-care ability was evaluated through the question, “Your current self-care ability is:”; possible answers were “complete”, “partial”, and “unable”. Compete self-care means that participants can complete ordinary daily activities on their own, without relying on others or support facilities. Partial self-care means that participants’ actions in daily life rely on auxiliary facilities such as walking sticks and wheelchairs. Being unable to rake care of oneself means full, daily reliance on others. And information about chronic disease is obtained by asking participant, “Have you been told by doctors that you have chronic disease”, possible answers were “yes”, and “no”.

The primary outcome variable, that is, quality of life of the empty-nest older adults, was measured using the Short Form 36-Item Health Survey (SF-36), which was developed in the United States and designed to allow self-evaluation of quality of life [19, 20]. This scale can be used directly and does not require license. It made up of 36 questions and two summary scores, namely, Physical and Mental Component Summary (PCS and MCS, respectively). The PCS encompassed the following four dimensions: physical function (PF), role physical (RP), bodily pain (BP), and general health (GH). The MCS contained the following four dimensions: vitality (VT), social function (SF), mental health (MH), and role emotional (RE). Individual item scores were summed up and transformed into a 0–100 scale, ranging from the worst to the best possible quality of life [21]. At present, SF-36 has been widely used to evaluate the quality of life of different older adults (community older people [22], rural older people [23], veterans [24], and senile hypertensive patients [25]) in China, and has been proved to have good reliability and validity. In this study, the scale’s Cronbach’s α was 0.751.

Questionnaires were administered at baseline and post-intervention (seven months). The questionnaire was completed following a face-to-face interview between an interviewer and a participant and collected on the spot. To ensure quality, completed questionnaires were checked carefully by quality supervisors after the interview. The response rate was 100%.

### Statistical analysis

EpiData was used for entering and checking the validity of data, and SPSS 22.0 software for statistical analysis. Data were expressed as mean ± SD (standard deviation). Difference between groups in terms of baseline characteristics was tested using chi-square test. The effect of the intervention versus control conditions was examined using ANCOVA analysis on post-intervention measurement values, controlling for pre-intervention scores. Cohen’s d was provided to evaluate the effect size with a guideline: trivial (< 0.20), small (0.20 to < 0.50), moderate (0.50 to < 0.80), and large (≥0.80) [26]. *P* value was statistically significant at (*P* < 0.05).

## Results

### Participants’ sociodemographic

Participants’ sociodemographic characteristics are shown in Table 1. Of the 350 participants, 190 (54.3%) were male and 160 (45.7%) were female; 244 (69.7%) were married and 16 (4.6%) had never been married; the age range was 60–88 years with a mean age of 69.96 ± 6.43 years. Meanwhile, 238 (68%) participants had a certain income every month, such as retirement pensions and supply from children or spouse, whereas 112 (32%) did not have a steady income. In addition, most participants graduated from secondary school or below (77.4%), were living with their spouse (60.9%), and completely self-cared (80.6%). No significant differences were found between intervention and control groups in terms of participant characteristics at baseline (*P* > 0.05).

**Table 1:** Comparison of the characteristics of the intervention and control groups

Characteristic	Total *N*(%)	Intervention	Control	*χ*^2^	*P*
		*n (%)*	*n (%)*		
**Sex**					
Male	190 (54.3)	96 (57.5)	94 (51.4)	1.317	0.251
Female	160 (45.7)	71 (42.5)	89 (48.6)		
**Age**					
60–69 years	175 (50.0)	86 (51.5)	89 (48.6)	1.865	0.394
70–79 years	135 (38.6)	59 (35.3)	76 (41.5)		
80 years or above	40 (11.4)	22 (13.2)	18 (9.9)		
**Education**					
No education	79 (22.6)	36 (21.6)	43 (23.5)	0.986	0.964
Primary school	95 (27.1)	47 (28.1)	48 (26.2)		
Secondary school	97 (27.7)	48 (28.7)	49 (26.8)		
High school	54 (15.4)	26 (15.6)	28 (15.3)		
Junior college	10 (2.9)	4 (2.4)	6 (3.3)		
University or above	15 (4.3)	6 (3.6)	9 (4.9)		
**Marital Status**					
Married	244 (69.7)	113 (67.7)	131 (71.6)	0.948	0.814
Never married	16 (4.6)	9 (5.4)	7 (3.8)		
Divorced	10 (2.9)	5 (3.0)	5 (2.7)		
Widowed	80 (22.8)	40 (23.9)	40 (21.9)		
**Living form**					
Living with spouse	213 (60.8)	101 (60.5)	112 (61.2)	0.919	0.821
Living alone	78 (22.3)	39 (23.3)	39 (21.3)		
Living with parents	50 (14.3)	24 (14.4)	26 (14.2)		
Living in a nursing home	9 (2.6)	3 (1.8)	6 (3.3)		
**Children visit frequency**					
Never visit	4 (1.1)	2 (1.2)	2 (1.1)	45.732	< 0.001
Irregularly	3 (0.9)	1 (0.6)	2 (1.1)		
Once more than half a year	7 (2.0)	4 (2.4)	3 (1.6)		
Once every six months	108 (30.9)	51 (30.5)	57 (31.2)		
1–2 times a month	113 (32.3)	52 (31.1)	61 (33.3)		
Once a week	52 (14.8)	29 (17.4)	23 (12.6)		
More than once a week	63 (18.0)	28 (16.8)	35 (19.1)		
**Employment status**					
Working	35 (10.0)	18 (10.8)	17 (9.3)	0.215	0.643
Not working	315 (90.0)	149 (89.2)	166 (90.7)		
**Income Source**					
Retirement pensions	127 (36.3)	64 (38.3)	63 (34.4)	2.665	0.751
Personal labor income	58 (16.6)	23 (13.8)	35 (19.1)		
Child supply	108 (30.9)	54 (32.3)	54 (29.5)		
Spouse supply	20 (5.7)	9 (5.4)	11 (6.0)		
Social relief	32 (9.1)	14 (8.4)	18 (9.9)		
Other	5 (1.4)	3 (1.8)	2 (1.1)		
**Monthly income**					
No income	112 (32.0)	51 (30.5)	61 (33.3)	1.922	0.589
< 1000 RMB	88 (25.1)	43 (25.8)	45 (24.6)		
1000–3000 RMB	90 (25.7)	40 (23.9)	50 (27.3)		
> 3000 RMB	60 (17.2)	33 (19.8)	27 (14.8)		
**Spousal relationship**					
Perfect	125 (38.6)	62 (40.5)	63 (36.8)	2.824	0.419
Good	170 (52.5)	79 (51.6)	91 (53.2)		
Bad	25 (7.7)	9 (5.9)	16 (9.4)		
Worst	4 (1.2)	3 (2.0)	1 (0.6)		
**Relationship with their children**					
Perfect	119 (35.6)	58 (36.7)	61 (34.7)	0.787	0.853
Good	188 (56.3)	86 (54.4)	102 (57.9)		
Bad	22 (6.6)	12 (7.6)	10 (5.7)		
Worst	5 (1.5)	2 (1.3)	3 (1.7)		
**Social activity participation**					
Most	37 (10.6)	19 (11.4)	18 (9.8)	1.419	0.701
More	117 (33.4)	60 (35.9)	57 (31.2)		
Less	154 (44.0)	69 (41.3)	85 (46.4)		
No	42 (12.0)	19 (11.4)	23 (12.6)		
**Self-care ability**					
Completely self-care	282 (80.6)	136 (81.4)	146 (79.8)	0.153	0.696
Partly self-care	68 (19.4)	31 (18.6)	37 (20.2)		
**Chronic disease**					
Yes	109 (31.1)	44 (26.4)	65 (35.5)	3.425	0.064
No	241 (68.9)	123 (73.6)	118 (64.5)		

***Note*:** The survey of the spousal relationship did not include the participants who had never married or were divorced, and the survey of their relationships with their children did not include participants without children

### Effects of the SMG-based intervention

The mean scores on the SF-36 for intervention and control groups at baseline and after the seven-month follow up are shown in Table 2. Overall, after controlling for pre-intervention assessment values, the post-intervention scores on MCS (*F* = 105.146, *P* < 0.001, Cohen’s *d* = 0.62) and PCS (*F* = 73.922, *P* = 0.011, Cohen’s *d* = 0.32) for intervention and control groups were significantly different. The scores of MCS and PCS increased significantly in the intervention group. Yet, for the eight subscales of QOL, the score changes of each dimension were different. For mental components, the scores on VT (*F* = 35.404, *P* < 0.001, Cohen’s *d* = 0.35), SF (*F* = 112.945, *P* < 0.001, Cohen’s *d* = 0.53), MH (*F* = 76.223, *P* < 0.001, Cohen’s *d* = 0.48) and RE (*F* = 71.127, *P* < 0.001, Cohen’s *d* = 0.62) for intervention and control groups were significantly different. For physical components, except for GH (*F* = 20.150, *P* < 0.001, Cohen’s *d* = 0.31), no significant differences in the scores on PF, RP, and BP were observed between the intervention and control groups.

**Table 2:** SF-36 scores at baseline and after 7 months with pre-intervention scores as a covariate

Variables	Group	Pre-intervention		Post-intervention			*F*	Cohen’s
		N	Mean ± SD	N	Unadjusted	Adjusted		
					Mean ± SD	Mean ± SD		
PF	Intervention	204	68.80 ± 22.53	167	68.92 ± 22.29	68.27 ± 0.12	0.405	0.05
	Control	192	67.54 ± 23.54	183	67.79 ± 23.24	68.38 ± 0.12		
RP	Intervention	204	55.24 ± 39.75	167	56.59 ± 39.60	54.40 ± 0.53	0.736	0.12
	Control	192	50.96 ± 39.08	183	51.78 ± 38.61	53.77 ± 0.51		
BP	Intervention	204	69.58 ± 16.38	167	71.24 ± 15.98	71.33 ± 0.69	2.429	0.08
	Control	192	69.78 ± 18.91	183	69.92 ± 18.92	69.84 ± 0.66		
GH	Intervention	204	57.02 ± 17.31	167	60.96 ± 17.71	60.94 ± 0.93	20.150*	0.31
	Control	192	56.98 ± 18.33	183	55.16 ± 19.20	55.17 ± 0.89		
VT	Intervention	204	61.23 ± 14.80	167	66.68 ± 14.57	66.77 ± 0.67	35.404*	0.35
	Control	192	61.45 ± 16.13	183	61.31 ± 16.15	61.23 ± 0.64		
SF	Intervention	204	68.49 ± 21.09	167	76.91 ± 20.74	75.80 ± 0.59	112.945*	0.53
	Control	192	66.17 ± 20.35	183	66.13 ± 20.25	67.14 ± 0.56		
RE	Intervention	204	59.28 ± 38.62	167	82.83 ± 34.68	81.59 ± 1.73	71.127*	0.62
	Control	192	56.10 ± 39.04	183	60.29 ± 38.46	61.43 ± 1.65		
MH	Intervention	204	64.50 ± 15.18	167	71.96 ± 15.05	71.99 ± 0.63	76.223*	0.48
	Control	192	64.57 ± 16.08	183	64.46 ± 16.09	64.43 ± 0.60		
PCS	Intervention	204	62.66 ± 17.74	167	66.68 ± 16.44	66.02 ± 0.36	73.922*	0.32
	Control	192	61.31 ± 18.45	183	61.16 ± 18.39	61.76 ± 0.34		
MCS	Intervention	204	63.38 ± 16.97	167	72.85 ± 14.55	72.30 ± 0.62	105.146*	0.62
	Control	192	62.07 ± 17.51	183	63.05 ± 17.20	63.55 ± 0.59		

***Note*:** ^*^P < .05

## Discussion

The main finding of this study was that in comparison to the control group, empty nesters who participated in the seven-month SMG-based intervention had significant improvement in quality of life. The mean scores for MCS, PCS, RE, VT, SF, MH, and GH among empty-nest older adults increased significantly. Moreover, these scores differed significantly from the corresponding scores in the control group.

In simplest terms, self-management describes what a person does to manage his/her disease [27]. A cross-sectional study [28] found that empty nesters’ awareness of self-health management is weak. Since empty nesters do not want their children to worry, they usually hide their illness and do not care about their health. They were also less likely to consult doctors [10]. These findings indicate that strong awareness of health responsibility may not have been formed among them. Therefore, the first stage of intervention is to help empty nesters develop self-health management awareness and ability. At the self-management stage, health responsibility is transferred to empty nesters themselves. Empty nesters are fully empowered as owners to understand and solve their own health problems with maximum self-efficacy. Previous studies have indicated a positive and significant relationship between social support and quality of life in the older adults [29, 30]. Specifically, Fatemeh et al. suggested that the older adults should be encouraged to participate in activities to improve their quality of life. In this study, mutual-management and group-management training were employed fully mobilize the enthusiasm of empty nesters to participate in social activities. Encouragement from peers can also inspire confidence in their continued participation. Owing to the particularity of empty nesters, it is difficult to achieve the expected results by a single management method. Therefore, this study divided health management into three stages to implement the intervention progressively. The series of measures, from individual to team, ensured the effectiveness of the full intervention.

Several studies have found that good social relationships (including relationships with relatives, neighbors, and friends) are the most commonly reported factor influencing quality of life in the older adults [31, 32]. After retirement, the older people usually return to their families and thus their professional and social roles are weakened. According to family theory, traditional living in their twilight years with their children is a good choice for the Chinese older people. But for empty-nest older adults, the absence of their children makes them lose part of social support and emotional communication [33]. They may doubt their existence and fall easily into a boring, helpless state. Therefore, in mutual-management and group-management stages, the emotional support provided by group members could meet their emotional needs to a certain degree. An offer of help from neighbors or friends could also alleviate their risk of being excluded from society. Moreover, study staff members conducted home visits once every two weeks to provide sufficient support and encouragement. By responding actively to their needs, study staff members ensured that the empty nesters would not feel uneasy when asking others for help because of concerns about burdening others and being rejected. These measures helped participants eliminate bad mood and maintain a healthy psychological state, which contributed to the significant increase in their scores related to psychological dimensions, including VT, SF, MH, RE, and MCS.

A cross-sectional study in Shaanxi province showed that education has a major influence on the quality of life among the older adults in China [34]. For example, Lasheras et al. indicated that a low educational level is associated with unhappiness, poor social relationships, and poor self-assessed health among the older people [35]. Highly educated people usually have stronger awareness of health management and pay more attention to their health status. However, the participants of this study have a low educational level, and a half of the empty-nest older adults have educational level of primary school or below. Therefore, popularization of health-related knowledge is particularly important. In the intervention, we held a series of lectures about quality of life, grasped their actual needs, and provided the necessary help. In the second stage, a Health Management Consultation Day was held. Participants could consult about health knowledge related to their illnesses. In the third stage, we also held a meeting to share mutual-assistant experiences. At the meeting, highly educated empty nesters shared their experiences and knowledge with others and offered necessary help through life or emotional support. Based on the above, participants had comprehensive understanding of their health and began to pay attention to having a healthy lifestyle. Meanwhile, at the end of the intervention, many empty nesters became friends with each other and stated that they would later organize related activities together to make use of their spare time.

In this study, no positive main effect on PF, RP, and BP was observed among empty-nest older adults. For physical function, this study assessed mainly the impact of participants’ health on daily life, such as strenuous exercise, housework, and going up and down the stairs. For physical role, this study assessed the impact of health status on the completion of work or social activities. For bodily pain, this study assessed the extent of physical pain. The result indicated that intervention had limited effects on improving physical health, which may be related to the limited duration of intervention In this survey, we found that some empty nesters have one or more chronic diseases. Therefore, the intervention has limited effect on improving the chronic disease status of empty nesters in the short term. Previous studies have showed that chronic disease is among the significant risk factors affecting quality of life in the older adults [36, 37]. Chronic diseases not only impair the health status of empty nesters but also affect their social activities. High medical costs may also cause a huge psychological burden. In the survey, many participants with arthritis reported having difficulty climbing stairs and some hypertensive patients had been recommended not to take strenuous exercises. According to the results, participants’ mental health improved, but the interventions had limited effect on alleviating long-term physical pain. Additionally, the intervention duration was limited given that physical health and lifestyle changes might be long-term processes. Therefore, professional medical services are needed to improve the physical health of individuals in this frail population.

Although there was no significant improvement in other dimensions of PCS, such as PF, RP, and BP, the current results indicated that the scores of GH and PCS also increased significantly after the intervention. The GH dimension is a self-assessment of overall health, whereas the PCS encompassed physical component scores. Findings indicate that participants felt their health improved after the intervention, which could be attributed to subjectivity of their self-assessment. Therefore, in future studies, multi-point measurement should be considered to evaluate better the intervention process and outcomes.

### Limitations

There are some limitations in this study that must be acknowledged. First, the low literacy level and advanced age of the study population might have caused participants to misunderstand the questions or give inaccurate responses. Second, researchers were not blinded to group allocation and the final statistical analyses. Therefore, subjective biases might have occurred inevitably when researchers conducted face-to-face interviews with participants. This requires researchers to fully understand the purpose of the study and be familiar with the content of the study, so as to help the participants better understand and complete the survey. So, investigators need to be trained before investigation. Moreover, although the group allocation was not actively disclosed to participants during the research process, participants in the intervention group might have guessed what the researchers wanted, the intervention effects might have been exaggerated. In addition, studies have shown the potential value of using more than one measures in a trial [38]. However, this study used only the SF-36 to measure the main outcomes. Therefore, future studies can use different measurements to assess better the long-term effects of interventions on quality of life.

## Conclusion

Our study demonstrated that the SMG-based health management has a positive effect on improving quality of life of empty-nest older adults. It provided a new perspective on bettering the quality of life of empty nesters, thereby establishing an important theoretical and practical foundation for the improvement and promotion of the model. These results should be tested further in larger samples and different settings.

## Supplementary Information

**Additional file 1.** A Study on Quality of Life of the Empty-nest Older adults.

## Data Availability

The dataset analysed during the current study is available from the corresponding author on reasonable request.

## Abbreviations

SMG: Self-Mutual-Group
SF-36: Short Form 36-Item Health Survey
MCS: Mental Component Summary
PCS: Physical Component Summary
PF: Physical function
RP: Role physical
BP: Bodily pain
GH: General health
RE: Role emotional
VT: Vitality
SF: Social function
MH: Mental health
HRQOL: Health-related quality of life
