# Supplementary material for: Efficacy of the self-mutual-group model targeting quality of life improvement among empty-nest older adults in Taiyuan, China: an intervention study
Source: BMC Geriatr. 2021 Mar 25;21:209. doi: 10.1186/s12877-021-02155-4 (PMC7992987; doi:10.1186/s12877-021-02155-4)
Supplement: Supplementary file 1 — Additional file 1. A Study on Quality of Life of the Empty-nest Older adults. [file 12877_2021_2155_MOESM1_ESM.docx]

A Study on Quality of Life of the Empty-nest Older adults

Date:

Investigator code:

Questionnaire Number:

General information

1. where do you live now?

province city district

1. Sex
2. male ②female
3. Age

①60-69 ②70-79 ③80 and above

1. Educational Level

①no education ②primary school ③secondary school

④high school ⑤junior school ⑥university and above

1. Marital status

①married ②never married ③divorced ④widowed

1. Are you still working now?

①Yes ②No

1. Your main source of income

①retirement pensions ②personal labor income

④child supply ④spouse supply

⑤social relief ⑥other

1. Monthly income

①no income ②less than 1000 RMB

③1000-3000 RMB ④more than 3000 RMB

1. Who are you currently living with?

①living with spouse ②living alone

1. living with parents ④living in a nursing home
2. Children visit frequency

①never visit ②irregularly ③once more than half a year

④once every six months ⑤1-2 times a month

⑥once a week ⑦more than once a week

1. How is your relationship with your spouse?

①perfect ②good ③bad ④worst

1. How is your relationship with your children?

①perfect ②good ③bad ④worst

1. How do you rate your participation in social activities?

①most ②more ③less ④no

1. Your current self-care ability is:

①complete ②partial ③unable

1. Have you been told by doctors that you have chronic disease?

①Yes ②No

SF-36 Questionnaire

Please answer the 36 questions of the Health Survey completely, honestly, and without interruptions.

1. In general, would you say your health is:

①Excellent ②very good ③Good ④Fair ⑤Poor

1. Compared to one year ago, how would you rate your health in general now?

①Much better now than one year ago

②Somewhat better now than one year ago

③about the same

④Somewhat worse now than one year ago

⑤Much worse than one year ago

1. LIMITATIONS OF ACTIVITIES

The following items are about activities you might do during a typical day. You’re your health now limit you in these activities? If so, how much?

a. Do you engage in vigorous activities, such as running, lifting heavy objects, participating in strenuous sports.

①Yes, limited a lot. ②Yes, limited a little. ③No, not limited at all.

b. Do you engage in moderate activities, such as moving a table, sweeping, Tai Chi, doing simple gymnastics, etc?

①Yes, limited a lot. ②Yes, limited a little. ③No, not limited at all.

c. Do you engage in any simple daily life activity, such as grocery shopping, shopping, etc.

①Yes, limited a lot. ②Yes, limited a little. ③No, not limited at all.

d. Up a few stairs.

①Yes, limited a lot. ②Yes, limited a little. ③No, not limited at all.

e. Up one stair.

①Yes, limited a lot. ②Yes, limited a little. ③No, not limited at all.

f. Stoop, squat, bend.

①Yes, limited a lot. ②Yes, limited a little. ③No, not limited at all.

g. Walk 1500 meters above the distance.

①Yes, limited a lot. ②Yes, limited a little. ③No, not limited at all.

h. Walk 1000 meters

①Yes, limited a lot. ②Yes, limited a little. ③No, not limited at all.

i. Walk 800 meters.

①Yes, limited a lot. ②Yes, limited a little. ③No, not limited at all.

j. Bathing and dressing yourself.

①Yes, limited a lot. ②Yes, limited a little. ③No, not limited at all.

1. PHYSICAL HEALTH PROBLEMS:

During the past 4 weeks, have you had any of the following problems with your work or other regular daily activities due to your physical health?

a. Cut down the amount of time you spent on work or other activities.

①Yes ②No

b. Accomplished less than you would like.

①Yes ②No

c. Were limited in the kind of work or other activities.

①Yes ②No

d. Had difficulty performing the work or other activities (for example, it took extra effort).

①Yes ②No

1. EMOTIONAL HEALTH PROBLEMS:

During the past 4 weeks, have you had any of the following problems with your work or other regular daily activities as a result of any emotional problems (such as feeling depressed or anxious)?

a. Cut down the amount of time you spent on work or other activities.

①Yes ②No

b. Accomplished less than you would like.

①Yes ②No

c. Didn't do work or other activities as carefully as usual

1. Yes ②No
2. Emotional problems interfered with your normal social activities with family, friends, neighbors, or groups?

①Not at all ②Slightly ③Moderately ④Severe ⑤Very severe

1. How much bodily pain have you had during the past 4 weeks?
2. None ②Very mild ③Mild ④Moderate ⑤Severe ⑥Very severe
3. During the past 4 weeks, how much did pain interfere with your normal work (including both work outside the home and housework)?
4. Not at all ②A little bit ③Moderately ④Quite a bit ⑤Extremely
5. ENERGY AND EMOTIONS:

These questions are about how you feel and how things have been with you during the last 4 weeks. For each question, please give the answer that comes closest to the way you have been feeling.

a. You feel full of life.

①all of the time ②most of the time ③A good bit of the time

④Some of the time ⑤A little bit of the time ⑥None of the time

b. You are a sensitive person.

①all of the time ②most of the time ③A good bit of the time

④Some of the time ⑤A little bit of the time ⑥None of the time

c. Your mood is very bad, nothing can make you happy.

①all of the time ②most of the time ③A good bit of the time

④Some of the time ⑤A little bit of the time ⑥None of the time

d. Your mind is very calm.

①all of the time ②most of the time ③A good bit of the time

④Some of the time ⑤A little bit of the time ⑥None of the time

e. You are energetic.

①all of the time ②most of the time ③A good bit of the time

④Some of the time ⑤A little bit of the time ⑥None of the time

f. Your mood is low.

①all of the time ②most of the time ③A good bit of the time

④Some of the time ⑤A little bit of the time ⑥None of the time

g. You feel exhausted.

①all of the time ②most of the time ③A good bit of the time

④Some of the time ⑤A little bit of the time ⑥None of the time

h. You are a happy person.

①all of the time ②most of the time ③A good bit of the time

④Some of the time ⑤A little bit of the time ⑥None of the time

i You feel bored.

①all of the time ②most of the time ③A good bit of the time

④Some of the time ⑤A little bit of the time ⑥None of the time

1. During the past 4 weeks, how much of the time has your physical health or emotional problems interfered with your social activities (like visiting with friends, relatives, etc.)?

①all of the time ②most of the time ③Some of the time

④A little bit of the time ⑤None of the time

1. GENERAL HEALTH:

How true or false is each of the following statements for you?

a. I seem to get sick a little easier than other people.

①Definitely true Mostly true Not sure Mostly false Definitely false

b. I am as healthy as anybody I know.

①Definitely true Mostly true Not sure Mostly false Definitely false

c. I expect my health to get worse.

①Definitely true Mostly true Not sure Mostly false Definitely false

d. My health is excellent.

①Definitely true Mostly true Not sure Mostly false Definitely false
